# Wish you were here: How defaunated is the Atlantic Forest biome of its medium- to large-bodied mammal fauna?

**DOI:** 10.1371/journal.pone.0204515

**Published:** 2018-09-25

**Authors:** Juliano André Bogoni, José Salatiel Rodrigues Pires, Maurício Eduardo Graipel, Nivaldo Peroni, Carlos A. Peres

**Affiliations:** 1 Programa de Pós-Graduação em Ecologia, Universidade Federal de Santa Catarina, Florianópolis, Santa Catarina, Brazil; 2 Universidade de São Paulo, Escola Superior de Agricultura “Luiz de Queiroz”, Laboratório de Ecologia, Manejo e Conservação de Fauna Silvestre (LEMaC), Piracicaba, São Paulo, Brazil; 3 Departamento de Ecologia e Zoologia, Centro de Ciências Biológicas, Universidade Federal de Santa Catarina, Florianópolis, Santa Catarina, Brazil; 4 School of Environmental Sciences, University of East Anglia, Norwich, United Kingdom; 5 Departamento de Sistemática e Ecologia, Universidade Federal da Paraíba, João Pessoa, Paraíba, Brazil; Smithsonian Conservation Biology Institute, UNITED STATES

## Abstract

Mammals represent the largest-bodied elements of the world’s surviving megafauna and provide several key ecosystems services, yet their populations are often under steep decline throughout the tropics. Anthropogenic defaunation is one the most important contemporary threats to modern mammal faunas. Although the Atlantic Forest biome of South America shows several clear signs of defaunation, the extent to which this biome has lost its mammal fauna remains poorly understood. Here, we collate and analyze a comprehensive body of secondary data to quantitatively assess the spatial patterns of defaunation of all medium- to large-bodied Atlantic Forest mammals which were then classed by morpho-ecological traits. We used a Defaunation Index, which was scaled-up to the entire biome using kriging interpolation, to examine the integrity of site-specific mammal faunas. We further use environmental and socioeconomic predictors to explain the drivers of defaunation. Our results show high levels of defaunation (>0.5) for most of the Atlantic Forest. Apex predators, other carnivores, large-bodied mammals and large herbivores were among the most defaunated functional groups. Remaining native vegetation cover, forest fragment size, and the largest neighboring forest remnant were the main negative predictors of defaunation. We conclude that medium- to large-bodied Atlantic Forest mammals are under high levels of threat due to historical population losses that continue today. A conservation action plan thus becomes imperative to prevent this biome from becoming an even “emptier forest”, severely compromising patterns of diversity, ecological processes and ecosystem functioning.

## Introduction

Mammals serve critical functional roles in natural ecosystems, for example, as primary consumers, top-down regulators of prey populations [1–3], seed dispersal agents maintaining forest carbon stocks [4–5], and several other non-redundant ecosystem services [6]. In virtually all environments worldwide, large mammals are severely threatened by population declines [6–8]. The key drivers of species losses include overhunting, and habitat conversion, degradation and fragmentation, whereby remaining populations become increasingly isolated in ever smaller patches within human-modified landscapes [6, 9–10]. Building on seminal studies (e.g., [11–13]), Redford (1992) [14] coined the term "empty forests"–defined as those succumbing to widespread vertebrate losses and downstream effects including the failure of key mammal-mediated ecological processes. Defaunation is a key ecological issue that has only recently been given sufficient attention. Widespread evidence so far indicates depletion or deletion of large-bodied species prior to replacements by small- and medium-sized species. This community-level phenomenon (i.e., density compensation) increases the abundance of some species, which may counterbalance a population decline, extirpation, or absence of potential competitors [15], leading to cascading effects that can propagate through entire communities in many ecoregions [7, 10].

The Atlantic Forest of South America is one of the most endangered major ecoregions worldwide, with only 11.7% (~16,377,472 ha) of its original vegetation cover remaining, most of which consisting of highly disturbed forest remnants now smaller than 50 ha [16]. The Atlantic Forest is widely recognized as a megadiversity hotspot [17], but the degree to which this biome has been emptied of its large vertebrate fauna is poorly documented. There is a growing body of evidence showing the ecological consequences of mammal defaunation [18–21], which is aggravated in extinction-prone mammal populations induced by a combination of several factors, such as small geographic range, low population density, slow life histories, delayed weaning age, and large body size [22–24].

The most threatened mammalian orders include Primates, Pholidota, Lagomorpha, Perissodactyla, Cetartiodactyla, and Carnivora [23, 25]. However, there is a general consensus that most mammals experiencing local extinctions in otherwise suitable habitats are game species persecuted by hunters [26]. Species morpho-ecological traits can be used to cluster mammals into functional groups [27–28]. Although species richness depends on environmental factors and functional diversity depends on the evolutionary history of a given region [29], functional groups are generally defined as a set of species sharing similar responses to analogous environments or contributing similar roles to ecosystem process [30]. Functional diversity can therefore help us understand patterns of occupancy, and the role of biological communities in ecosystem functioning [31].

Although ample evidence of defaunation has been shown for several neotropical ecoregions (e.g., [32–33]), a more comprehensive biome-scale study can help us understand the spatial patterns of defaunation along the longest tropical forest latitudinal gradient worldwide. In doing so, this serves as a baseline comparison for each Atlantic Forest locality or provincial region and between other forest biomes both in the Neotropics and the Paleotropics. An understanding of the extent to which an ecosystem and its components are threatened is also critical to inform conservation strategies. Several studies at regional scales have shown that mammalian assemblages succumb to high levels of defaunation. However, the main drivers of defaunation and the degree to which this process affects different functional groups remain poorly understood for virtually all tropical mammal faunas at multiple biogeographic scales.

Here, we compiled published and unpublished secondary data from virtually all studies of medium to large-bodied mammals along the entire Atlantic Forest domain to (i) assess patterns of defaunation throughout this biome and for different mammal functional groups; (ii) interpolate these patterns of defaunation to surrounding areas to map the biome-wide spatial distribution of assemblage decay for different mammalian taxa; and iii) use environmental and socioeconomic variables to predict estimates of local defaunation. We hypothesize that levels of defaunation across the Atlantic Forest are elevated at regional to subregional scales and that more defaunated regions overlap densely-settled coastal zones, with decreasing values along large tracts of sparsely settled remaining montane forest along the Serra do Mar and Serra Geral regions. The functional groups expected to succumb to the highest local extinction rates include apex-predators such as large-bodied carnivores, followed by large herbivores and mesocarnivores. Finally, we expect the main predictors of defaunation at the local scale to include low landscape-scale native vegetation cover, wholesale habitat conversion (e.g. into agriculture, silviculture and urban settlements), elevated human population density and, analogous to the trend of Kuznets environmental curves [34], high levels of human prosperity as expressed by the human development index (HDI) and per capita income of neighboring municipal counties.

## Material and methods

### Ethics statement

Part of the data used in this study was authorized based on license number 47255 from Instituto Chico Mendes de Conservação da Biodiversidade (ICMBio). We confirm that the field studies did not involve handling of any endangered or protected species, but only species records via non-invasive sampling such as camera-trapping. This work was not submitted to an Institutional Animal Care and Use Committee (IACUC) or equivalent animal ethics committee, because the data were largely based on camera-trapping. Sampling procedures and/or experimental manipulations were reviewed or specifically approved as part of obtaining the specific field permit (license number 47255) which was issued by ICMBio.

### Mammalian assemblages and functional groups

**Fig 1:**
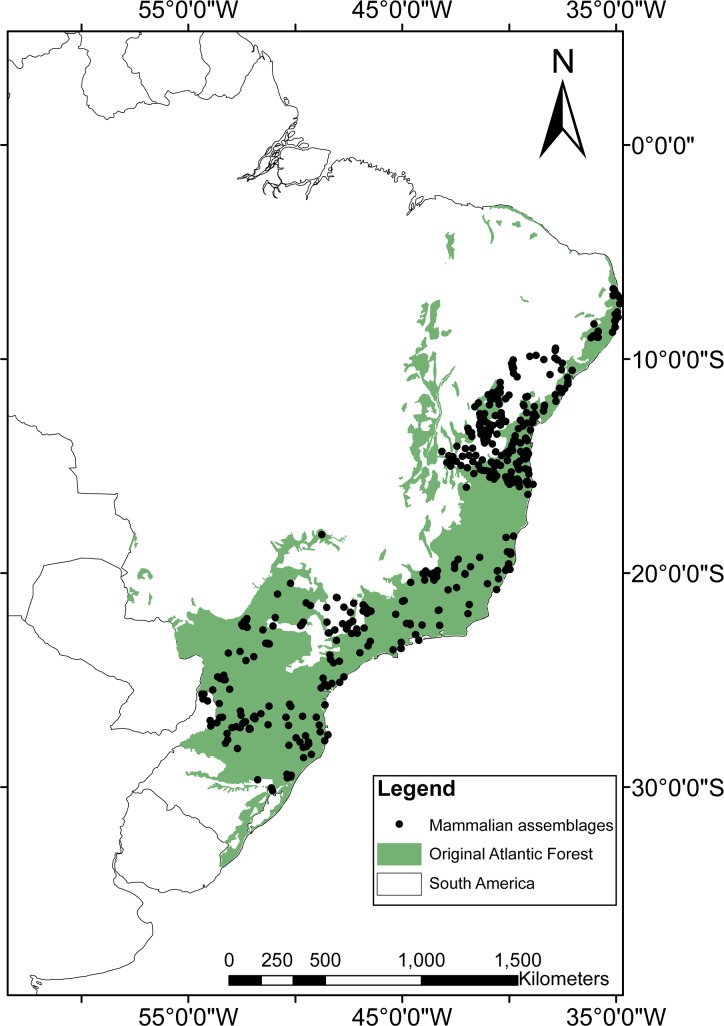
Spatial distribution of the 497 mammalian mammal assemblages across the Atlantic Forest biome of South America on which this study is based.

We use data on medium- and large-bodied (i.e. adult body mass ≥1 kg [35]) mammal assemblages throughout the entire Atlantic Forest biome, spanning parts of Brazil and Argentina, initially contained in Canale et al. (2012) [9], Bogoni et al. (2017) [36], and Lima et al. (2017) [37]. We further completed the dataset using search tools and the keywords (in English, Portuguese and Spanish) “*medium- to(and) large-sized(bodied) mammals*” and “*Atlantic Forest*” in ‘Scopus’, ‘Web of Science’ and ‘Scielo’, using the operator ‘AND’ to pursue different word combinations [36, 38]. We also replaced “mammals” with all major mammalian orders in the Atlantic Forest (e.g. “primates”, “carnivores”, “ungulates”). We then complemented this database with searches in ‘Google Scholar’, ‘Google’ and ‘ResearchGate’. All supplementary data used in this analysis were compiled between January and April 2018, and included unpublished dissertations and peer-reviewed studies that were published or *in press*, within the original extent of the entire Atlantic Forest (Fig 1). We included all studies for which mammal inventories had been carried out between 1983 and 2017. This compilation therefore captures the vast majority of available studies. Taxa that had not been identified to species level (e.g. limited to family or genus level) were not included in the presence-absence database. Our alpha taxonomy and estimates of adult body mass follow Paglia et al. (2012) [39]. Recent taxonomic arrangements concerning the distribution of the genus *Galictis* were solved following Bornholdt et al. (2013) [40] and the IUCN (2016) [41] distribution maps. The congener felids *Leopardus tigrinus* and *L*. *guttulus* were defined as closely related ecological analogues or ecospecies (i.e. *Leopardus* spp.), because they serve similar ecological roles [42–43]. Additional taxonomic revisions made during the study period did not affect our overall classification. Species associated with open habitat areas (i.e. *Conepatus* spp. and *Lycalopex* spp.) and nocturnal species with scansorial or arboreal habits (i.e. *Tamandua tetradactyla*, *Potos flavus* and *Coendou* spp.) were removed from the initial dataset because their contemporary occupancy could not always be reliably documented. The sloth *Bradypus torquatus* and the large rodent *Myocastor coypus* were not considered in this analysis because they are highly inconspicuous. Finally, we completed the dataset using all available evidence of records of three midsized to large primate genera (*Alouatta*, *Brachyteles* and *Sapajus*) on the basis of both the specialized literature and expert opinion (S1 File). In doing so, we first plotted the coordinates of all records of these primate genera and a 100-km radial buffer. Subsequently, the 497 mammal assemblages considered here were updated to include each of these primate genera on the basis of reliable records within the same landscape. For each study, we counted the number of field techniques that had been deployed in each mammal inventory and assessed the likelihood of recording arboreal/scansorial species given the survey methods employed. In addition, the geographic coordinates, dominant vegetation type, forest fragment sizes (ha), and elevation (masl) of each survey site were recorded. Missing data related to vegetation type, geographic coordinates and elevation were solved by combining the maps provided in each article or dissertation, with digital vegetation classification maps [44] and Google Earth (2015) [45] imagery. Vegetation physiognomy at each site was grouped into three broad mutually exclusive classes: (1) Dense Ombrophilous Forest (FOD), including lowland forest; (2) Mixed Ombrophilous Forest (FOM), including montane grasslands; and (3) Deciduous (or Semideciduous) Seasonal Forest (FED), including dry forest.

Based on morpho-ecological traits, all mammal species were classified into 10 trophic guilds or functional groups, which were not necessarily mutually exclusive. In this trophic guild classification, we ranked the energetic stratum of modal dietary patterns, as following: (1) folivore < (2) frugivore < (3) granivore < (4) insectivore < (5) myrmecophage < (6) mesocarnivore < (7) hypercarnivore. We then weighted the proportion of each major dietary mode of any given species (sourced from Wilman et al., 2004 [46]) by these energetic levels (e.g. if an *Alouatta* population consumes 80% leaves and 20% fruits, its trophic level would be 1.2 (i.e. (0.8 × 1) + (0.2 × 2)). Mammal species were assigned to the following functional groups: (1) frugivores; (2) large grazers or browsers: e.g. genus *Mazama*, *Ozotoceros*, *Hydrochoerus* and *Tapirus*; (3) mesocarnivores (body mass <13kg); (4) apex carnivores (>13kg); (5) small-bodied species (<10kg); (6) large-bodied species (≥10kg); and (7) megafauna (> 44kg). These body mass thresholds and broad trophic classes are based on Martin and Klein (1995) [47], Wilman et al. (2004) [46], Roemer et al. (2009) [2], Paglia et al. (2012) [39], and Wallach et al. (2015) [48]. Overall conservative biomass estimates for each mammalian assemblage were computed based on the sum of mean adult (male and female) body mass of each species contained in each assemblage, based on the presence-absence matrix.

### Diversity, defaunation index and spatial interpolation

We quantified for each site key descriptors of diversity, including total species richness and richness of functional groups [49]. We calculated a defaunation index (*sensu* Giacomini and Galetti, 2013 [33]) for both the entire mammal assemblage (total defaunation) and for each functional group (assemblage-level). To avoid Type I errors (i.e., pseudo-absences), we also calculated the defaunation index for each metacommunity, based on mammal assemblages in any given cluster within a 50-km radius of each other (cluster-level). Clusters were defined taking into account the average home range size of the mammalian fauna recorded (~788.4 km^2^ based on Jones et al. 2009 [50]), multiplied by three (following Maffei and Noss 2008 [51]).

The defaunation index is a weighted measure of dissimilarity between the contemporary mammal assemblage and a reference assemblage representing a historical and/or faunally intact or undepleted baseline. This index ranges from 0.0 (completely intact) to 1.0 (completely defaunated) and is based on the Bray-Curtis dissimilarity index with some modifications (see Giacomini and Galetti, 2013 [33]). To calculate this index, we used a baseline assemblage assuming probable occurrences on the basis of known geographic range polygons obtained from the IUCN (2016) [41] to determine the historical presence of each species at each of the 497 sites and 164 mammal metacommunities. Obtaining mammalian presence data from IUCN polygons has become a widely established methodology in the conservation ecology literature (e.g., [52–53]). However, we accept that the IUCN range maps are based on limited available information, rather than true local occupancy data, and the potential number of species at any given site can be overestimated. For continental-scale studies this is the only available dataset, which is now a standard data source in highly cited papers and international databases available for all terrestrial mammals [52–53]. To mitigate this potential problem, we adjusted the defaunation index by decreasing each value for both scales of analysis (i.e., assemblage-level and cluster-level) by 20% based on either baseline matches (71%) or omission errors (9%) for distribution maps of amphibian species of Mesoamerica, that together aggregates to a confidence level of 80% [54]. Matches represent species that are both reported by inventories and included in IUCN polygons, whereas omission error represent species reported as missing in the polygons but detected by any given inventory [54]. Although these data are virtually unavailable for mammals, amphibian geographic ranges can be a good calibrator in this case, due to the fact that their distribution is on average ~35 times more restricted than that of mammals, thereby intrinsically increasing the probability of omissions or Type I errors [54].

For the historical baseline, we further adjusted the IUCN range polygons of species that had been extirpated at regional or subregional scales, or that had their distribution areas significantly reduced, on the basis of credible reconstructions of their historical range. These included *Panthera onca* [55], *Ozotoceros bezoarticus* [56], *Priodontes maximus* [57], *Pteronura brasilienses* [58] and *Brachyteles* spp. [59–60]. We examined levels of defaunation in terms of the species importance (D_bs_) value (ω), defined as an intrinsic feature that distinguishes this index from the usual Bray-Curtis index based on a species trait (e.g. body size). In representing ω, we assigned adult body mass (obtained from Paglia et al., 2012 [39]) elevated to the ¾ power to account for the metabolic allometry of different species as a function of body size [33, 61]. Moreover, we also explored descriptors of diversity for historical mammal assemblages by overlaying the modern and historical assemblages to explore the incidence of local extinctions per biogeographic province (which were segmented based on the main hydrographic basins of the Atlantic Forest).

To interpolate defaunation estimates at the assemblage level for the entire Atlantic Forest biome, we initially used the Moran Index (M) to assess the spatial autocorrelation of mammal species richness [62]. Since spatial autocorrelation was detected, we used a kriging approach to interpolate the final defaunation map [63–65]. Kriging, which is frequently used for optimal data interpolation, is an inverse distance weighting (IDW) geostatistical method that requires a semivariogram model to describe the spatial autocorrelation pattern of any particular variable [64–65]. We adopted an interpolation approach to show levels of defaunation at subregional to biome-wide scales in terms of (i) how local contemporary mammal assemblages represent a nested subset of the historical species richness and composition [36, 38]; and (ii) how the historical and regional patterns of human occupation and degradation throughout the Atlantic Forest may converge across subregional provinces [16, 66], likely predicting population declines and local extinctions [7, 67].

### Socioeconomic and land use context

Based on the geographic coordinates of each site, expressed as a UTM projection (Datum WGS 84), we extracted data on the human development index (HDI: United Nations Development Program) within a 10-km buffer area (~31,060 ha) around each site using the mean area-weighed HDI and per capita income (USD) values for all neighboring municipal counties, on the basis of the Brazilian Atlas of Human Development [68]. These buffers were defined as five times the buffer area used to assess the effects of landscape cover on mammalian carnivores in the Atlantic Forest [69]. Next, based on the ‘SOS Mata Atlântica’ land cover maps [70], assuming the 2016 landscape cover as a proxy for all studies, we extracted the following land cover data for each 10-km buffer area: (1) native vegetation cover (NC): sum of all natural vegetation types; (2) largest fragment area (LF): largest remnant of natural vegetation; (3) anthropogenic habitat cover (AC): sum of all areas allocated to agriculture, livestock pastures and exotic tree plantations (e.g. *Pinus* spp. and *Eucalyptus* spp.); (4) total urban area (UA); and (5) open water (W), including both freshwater and marine environments. We also obtained the total human population of each county (human population [HP]) that entirely or partially overlapped each 10-km buffer, based on data from the Brazilian Institute of Geography and Statistics [71]. Buffer areas outside the phytogeographic boundaries of the Atlantic Forest (e.g. Atlantic Forest/Cerrado ecotone) were excluded due to missing data in the ‘SOS Mata Atlântica’ land-use maps. All geographic data extraction was conducted using the MapInfo 11.0 software [72].

### Predictive analysis

**Fig 2:**
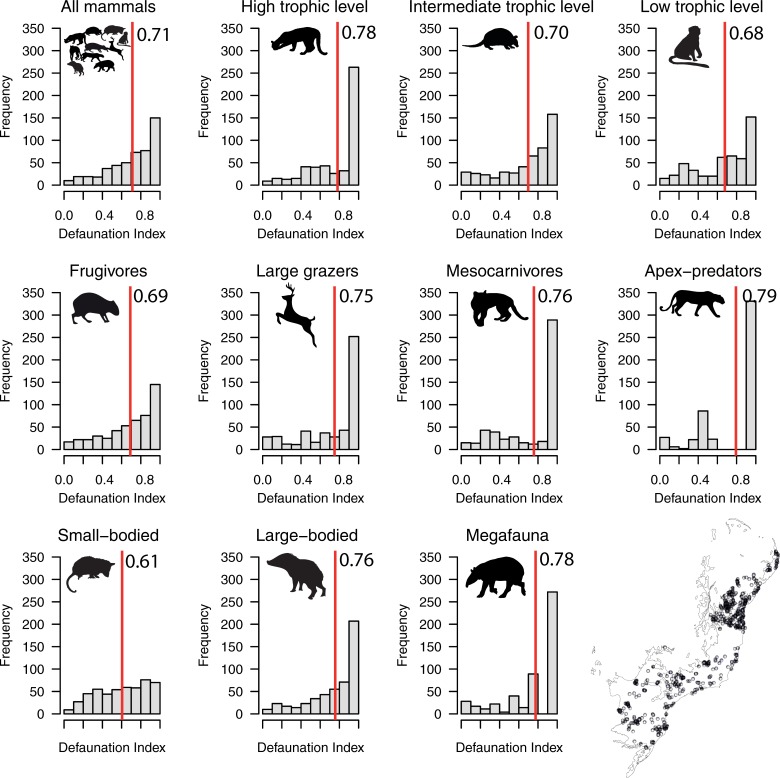
Frequency distribution of the overall defaunation index for medium- to large-bodied mammals at 497 study sites across the Atlantic Forest biome of South America. Red lines indicate mean values. Inset map (lower right) shows the geographic distribution of Atlantic Forest mammal assemblages compiled in this study.

Incorporating only study sites associated with a complete set of predictors (e.g. site and landscape metrics), we fitted linear regression models to predict the extent to which assemblage-level defaunation had taken place [73–74]. We did not perform regression models at the cluster scale given that local to landscape explanatory variables were obtained at the assemblage level. Models were defined a priori—independently of all possible combinations—according to our goals of assessing the relative contribution of predictors to levels of defaunation, including: (i) methodological noise; (ii) landscape features; (iii) socioeconomic indices; and (iv) the combination of both landscape and socioeconomic factors. Bivariate and multiple regression models—with a maximum of four covariates examined together to avoid high variance inflation factors (VIF > 10)—were performed using fragment size (ha), dominant vegetation type (FOD, FOM and FED), elevation (masl), and the number of field survey techniques used, whether or not arboreal/scansorial species had been recorded. We also considered HDI, human population density, the largest neighboring fragment area, and land cover type within 10-km buffers (native vegetation, anthropogenic habitats, urban areas and water bodies). Models were performed using a gamma distribution due to: (i) non-normality of defaunation indices examined a priori using Shapiro-Wilk tests and histograms; and (ii) defaunation histograms, which showed an overdispersed distribution, typically >0.7 [73] (see Fig 2). To define the ‘best’ predictive model(s) we used AIC values and pseudo-r^2^ which were obtained from 1-(residual deviance/null deviance). Models with ΔAIC < 4.00 were also considered plausible as the ‘best’ model among all candidates. Model intercepts showed regression trends and model significance was based on a chi-square test adapted to evaluate differences between null deviances and residual deviances in relation to model degrees of freedom [73]. Model post-validation was based on overdispersion values (OD <0.5), VIF < 10, variance diagnostic plots and dispersion parameters for the error structure used [73–74]. In the regression plots, we used the “loess” method to adjust regression curves and their respective 95% credible intervals, and two curves were added based on a threshold obtained from average predictors [75]. All statistical analyses were performed in R [76] using the *ade4* [77], and *gstat* packages [64].

## Results

### Defaunation estimates and spatial interpolation

**Fig 3:**
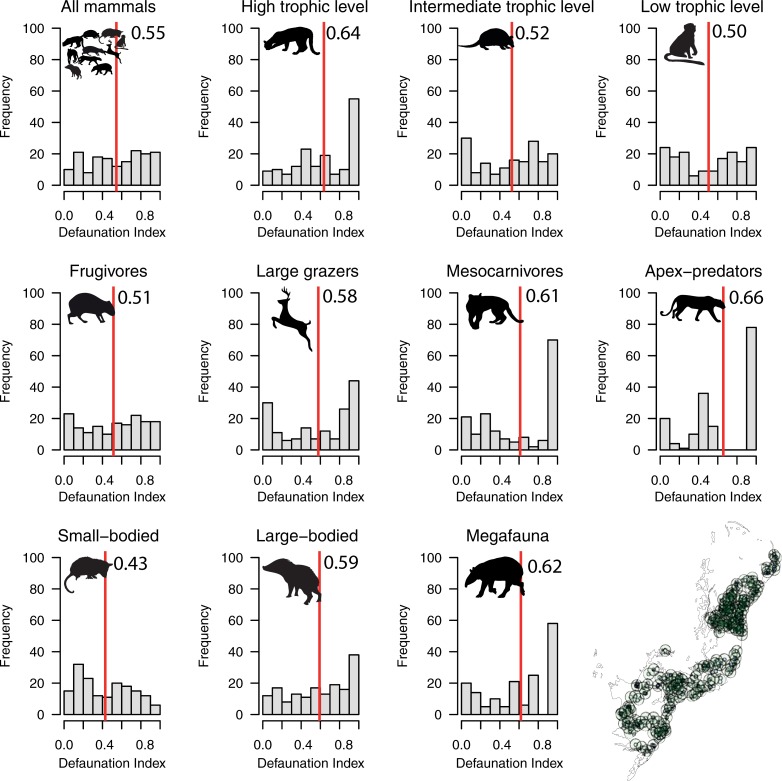
Frequency distribution of the overall defaunation index for medium- to large-bodied mammals at 497 study sites grouped into 164 clusters of 50 km in radius (referred to as ‘metacommunities’) across the Atlantic Forest biome of South America. Red lines indicate mean values. Inset map (lower right) shows the geographic distribution of Atlantic Forest mammal assemblages compiled in this study.

In total, we obtained data from 497 mammal assemblages distributed throughout the Atlantic Forest from a total of 105 studies that were consistent with our selection criteria (Fig 1). This resulted in 164 independent clusters of study sites from which mammal inventories are available (Fig 2; Fig 3; S2 File). In total, we compiled 3,522 presence records representing 41 mammal species in the contemporary occurrence dataset. On average, 7.1 species were recorded per site (range = 0–28 species and 0–17 species within any given functional group) (S1 Fig), and mammal species richness was spatially autocorrelated across all sites (M_obs_ = 0.19; M_exp_ = -0.01; p < 0.01). Considering the baseline historical dataset, a maximum of 34 mammal species co-occurred at any given site (mean = 25.9 species per site), indicating an average reduction from historical to modern times of 72.5% in terms of species richness and 80.5% in terms of overall biomass. Comparing contemporary and historical mammal assemblages at any given site for all mammal taxa yielded a mean total defaunation index of 0.71 (± 0.25). Adjusting the defaunation index according to a historical baseline based on an overall 20% overestimation of existing mammal distribution polygons, the mean overall index was 0.57 (±0.20). Among individual functional groups, defaunation estimates ranged from 0.61 for small-bodied species to 0.79 for apex-predators. Considering independent clusters of sites, we obtained a mean total defaunation index of 0.55 (± 0.29), ranging from 0.43 in small-bodied species to 0.66 in apex-predators. Our cluster-based defaunation estimate was therefore 22.5% lower than that for individual assemblages. While adjusting the defaunation index based on an overestimation of 20% in mammal distribution polygons, the index for cluster-level defaunation was 0.44 (±0.23), representing a decrease of 38% compared to the worst-case scenario (i.e. 0.71 for total unadjusted defaunation index (Dbs) at the assemblage level). Yet defaunation values were typically higher than 0.5, except for small-bodied species at the cluster-level (Fig 2 and Fig 3). The highest defaunation values were estimated for apex-predators (0.79 and 0.66 at the assemblage and cluster-level, respectively), mammals feeding at highest trophic levels [i.e. ≥5, myrmecophages and carnivores] (0.78; 0.64), all megafauna (0.78; 0.62), all large-bodied species (0.76; 0.59), and all large herbivores (0.75; 0.58) (Fig 2 and Fig 3). Approximately 59% of the 497 sites had experienced higher than average defaunation levels, particularly for apex-predators (66.6%), mesocarnivores (63.2%), species at intermediate trophic level (61.6%), species at high trophic levels (60.4%), large herbivores (60.2%), and large-bodied species (59.6%) (Fig 2 and Fig 3; Table 1). These defaunation values at the assemblage-level were higher than 54% (except for small-bodied mammals) even if the original values were reduced by 20% (Table 1: Dbs_adj_).

**Table 1:** Defaunation index (Dbs) and adjusted defaunation index for different mammal trophic levels and functional groups across the entire Atlantic Forest biome (at both the assemblage- and cluster scales) and Dbs broken down by provincial regions at the assemblage-level only.

	Defaunation values at the assemblage- level			Defaunation values at the cluster-level					Defaunation (Dbs) values (by Provinces at the assemblage-level)			
Approach	Dbs (±SD)	Dbs_adj_ (±SD)	Sites above average* (N in 497 and %)	Dbs (±SD)	Dbs_adj_ (±SD)	Cluster above average* (N in 164 and %)	EA	NA	PR	SA	SEA	UR
Total	0.71 (0.25)	0.57 (0.20)	293 (59.0)	0.55 (0.29)	0.44 (0.25)	84 (51.2)	0.85	0.90	0.62	0.58	0.49	0.66
High trophic level	0.78 (0.27)	0.62 (0.22)	300 (60.4)	0.64 (0.32)	0.51 (0.27)	84 (51.2)	0.90	0.95	0.70	0.65	0.57	0.75
Intermediate trophic level	0.70 (0.30)	0.56 (0.24)	306 (61.6)	0.52 (0.32)	0.42 (0.27)	90 (54.9)	0.80	0.90	0.62	0.61	0.48	0.69
Low trophic level	0.68 (0.29)	0.54 (0.23)	291 (58.6)	0.50 (0.33)	0.40 (0.28)	86 (52.4)	0.86	0.84	0.56	0.47	0.44	0.51
Frugivores	0.69 (0.27)	0.55 (0.22)	289 (58.1)	0.51 (0.31)	0.41 (0.26)	89 (54.3)	0.83	0.89	0.60	0.56	0.46	0.63
Large grazers	0.75 (0.32)	0.60 (0.25)	299 (60.2)	0.58 (0.37)	0.46 (0.31)	90 (54.9)	0.86	0.96	0.66	0.68	0.57	0.7
Mesocarnivores	0.76 (0.32)	0.61 (0.26)	314 (63.2)	0.61 (0.38)	0.49 (0.33)	86 (52.4)	0.97	0.86	0.64	0.39	0.5	0.61
Apex-predators	0.79 (0.31)	0.63 (0.25)	331 (66.6)	0.66 (0.36)	0.53 (0.31)	78 (47.7)	0.89	0.98	0.72	0.75	0.59	0.81
Small-bodied	0.61 (0.27)	0.48 (0.22)	256 (51.5)	0.43 (0.28)	0.34 (0.24)	78 (47.7)	0.79	0.77	0.51	0.34	0.34	0.46
Large-bodied	0.76 (0.27)	0.61 (0.22)	296 (59.6)	0.59 (0.32)	0.47 (0.27)	87 (53.1)	0.87	0.95	0.66	0.69	0.55	0.73
Megafauna	0.78 (0.30)	0.62 (0.24)	272 (54.7)	0.62 (0.36)	0.49 (0.31)	83 (50.1)	0.91	0.97	0.64	0.71	0.56	0.78

Provinces: EA: East Atlantic; NA: Northeast Atlantic; PR: Paraná; SA: South Atlantic; SEA: Southeast Atlantic; UR: Uruguay.

* Refers to Dbs values.

**Fig 4:**
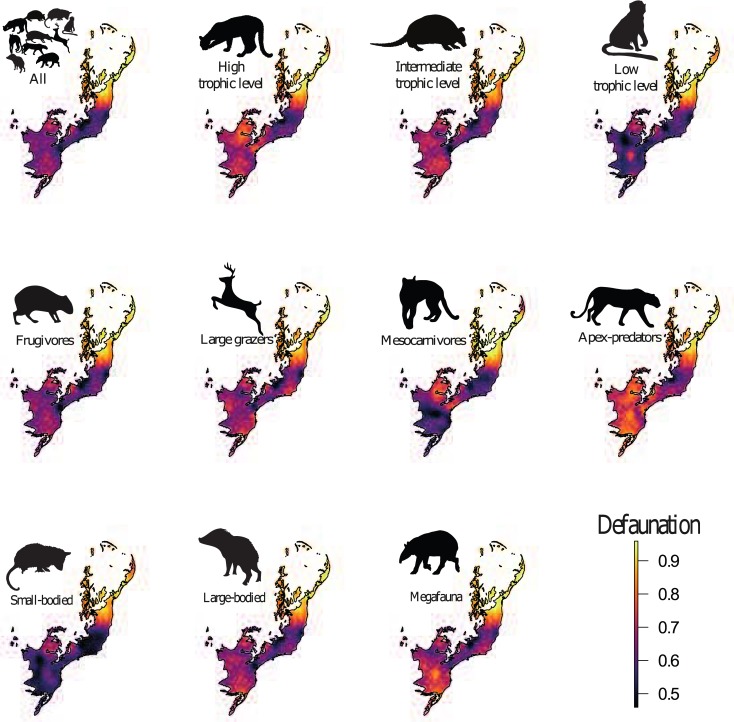
Spatial kriging interpolation of the defaunation index of medium- to large-bodied mammal species across the Atlantic Forest of South America. The color gradient of defaunation ranges from most intensive in yellow and least intensive in blue.

**Fig 5:**
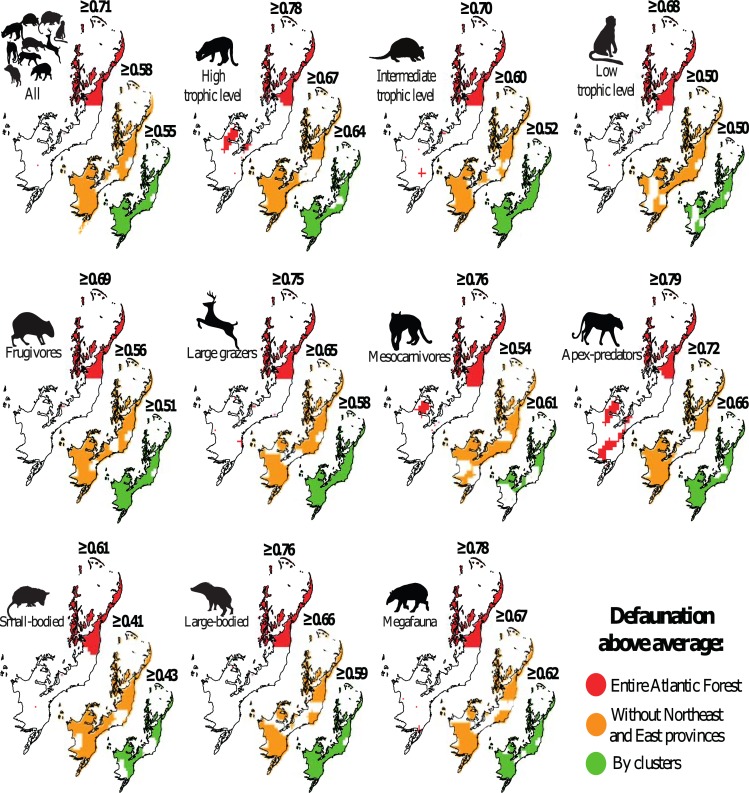
Geographic distribution of areas showing larger-than-average defaunation indices (based on kriging interpolation) for medium- to large-bodied mammal species across the Atlantic Forest biome. Red represents above-average assemblage-level defaunation; orange above-average defaunation excluding the Northeast and East provinces; green represents above average defaunation at the cluster-level.

**Fig 6:**
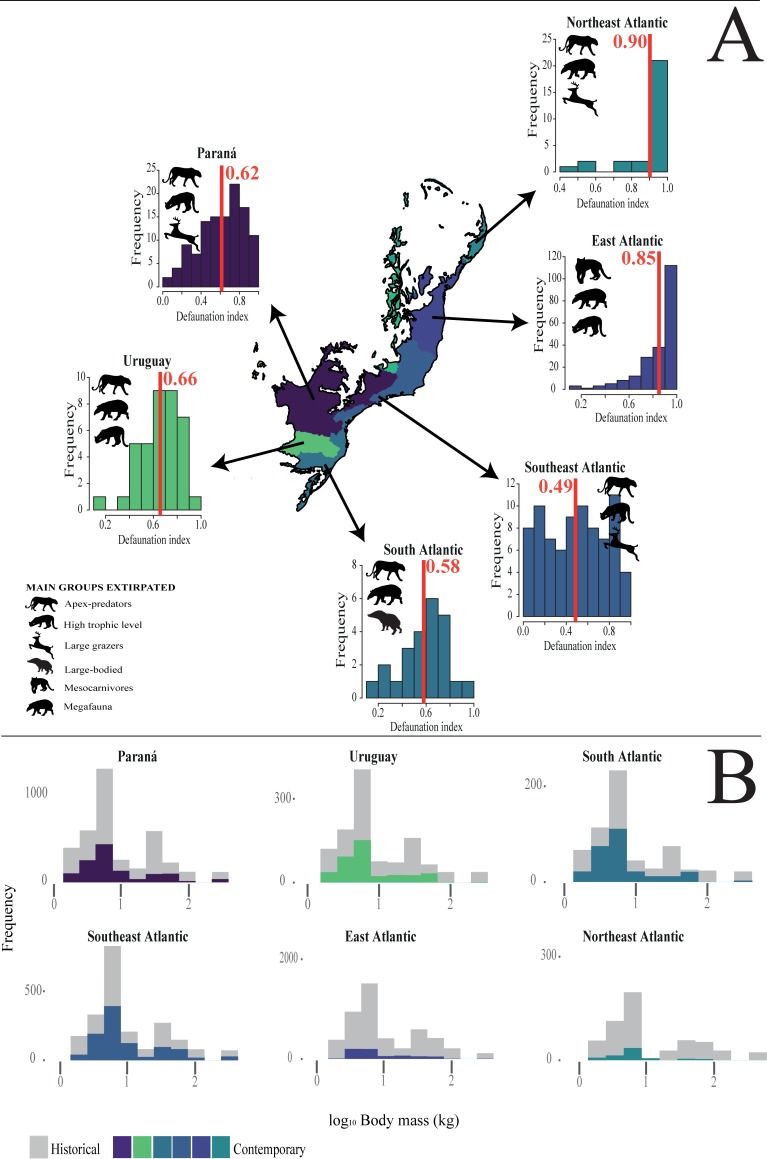
**(A) Provincial scale distribution of the overall defaunation index of medium- to large-bodied mammals of the Atlantic Forest of South America.** Red lines indicate mean values; **and (B) Comparisons between the historical and contemporary distributions of mammal assemblage biomass estimates for different Atlantic Forest provinces.** Gray and colored bars represent the historical biomass and the contemporary biomass, respectively, at each province within the Atlantic Forest biome.

Kriging interpolation surfaces showed that the functional groups succumbing to the highest local extinction rates compared to historical distributions included apex-predators, species at high trophic levels, and large-bodied species (Fig 4) but this varied widely geographically among functional groups (Fig 5). Regions experiencing the highest levels of defaunation are concentrated in the eastern portions of the Atlantic Forest (Northeast and East Atlantic provinces), ranging from the states of Pernambuco to northern Minas Gerais. In the Northeast and East Atlantic provinces, all mammal functional groups exceeded the average defaunation for the entire Atlantic Forest. Excluding these two provinces, the average defaunation estimate for the entire Atlantic Forest was 0.58, representing a reduction of 18.3% in relation to all mammal assemblages. We also observed high levels of defaunation in the western portions of the southern Atlantic Forest, ranging across western Rio Grande do Sul, and the southern Brazilian states of Santa Catarina and Paraná (i.e. 25° - 30°S; 50° - 55°W; Uruguay and Paraná provinces), where 80% of all mammal functional groups exceeded the defaunation average for the entire Atlantic Forest. Coastal regions (15° - 22.5°S; 37.5° - 45°W and 10° - 12.5°S; 37.5° - 40°W; Southeast Atlantic province) were less defaunated, with only one functional groups (apex-predators) exceeding the biome-wide average of 0.58. In the central region of the Atlantic Forest (20° – 22.5° S; 50° - 55°W; Paraná province), 60% to 80% of all functional groups were defaunated above the regional average (Figs 4, 5 and 6). In contrast, high-elevation areas of the Serra do Mar and Serra Geral–which span a montane knife-ridge from the states of Rio de Janeiro to Rio Grande do Sul–were the least defaunated across the entire biome (Figs 4, 5 and 6).

### Predictors of defaunation

**Fig 7:**
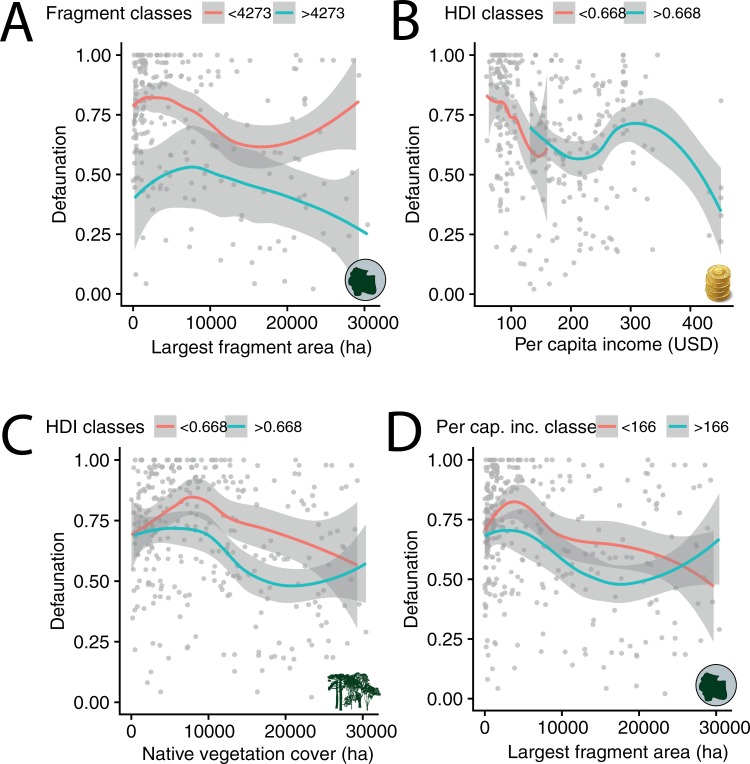
**Overall defaunation index of medium- to large-bodied Atlantic Forest mammals in relation to (A) the area (ha) of native vegetation cover within a buffer of 31,060 ha; (B) anthropogenic habitat cover; (C) area (ha) of the largest neighboring forest remnant; (D) and elevation (masl).** Curves represent the best fit of regression models based on the loess method. Two curves were added based on a threshold obtained from average predictors (i.e. below and above average in orange and green, respectively).

Of the 497 mammal assemblages in our database, we obtained a core number of covariates to fit overall defaunation models for 317 (63.8%) assemblages. On average, overall defaunation of these 317 assemblages was 0.69 (± 0.26). The main predictors of total defaunation in a multivariate model included local forest patch size and area of the largest available forest remnant (AIC = 96.21; p = 0.02; pseudo-r^2^ = 0.16) and three other candidate models with ΔAIC < 4.00 (Table 2; Fig 7). Models at the landscape scale, however, included significant effects of native vegetation cover and water bodies. Among single models, we found significant predictors of defaunation in the number of field survey techniques used (AIC = 109.0; p = 0.02; pseudo-r^2^ = 0.11), and native vegetation cover (AIC = 130.7; p = 0.06; pseudo-r^2^ = 0.05) (Table 2).

**Table 2:** Bivariate and multiple regression models predicting overall defaunation of medium- to large-bodied mammal assemblages across the Atlantic Forest biome.

Model	Type	AIC	ΔAIC	Int.	k	pseudo-r^2^	Sig. null	DF	OD	VIF
**FS + LF** *	Landscape	96.21	0	-0.10	2	0.16	**0.02**	223	0.21	<2
**FS + NC + LF** *	Landscape	96.67	0.46	-0.10	3	0.16	**0.03**	223	0.21	<8
**FS + NC + LF + W** *	Landscape	98.61	2.40	0.00	4	0.16	0.07	223	0.20	<9
**NT + AS** *	Methodological	99.47	3.26	-0.11	2	0.14	**0.01**	316	0.20	<2
NT	Methodological	109.0	12.83	0.74	1	0.11	**0.02**	316	0.21	-
HDI + NC	Social + Landscape	118.0	21.80	-0.10	2	0.09	**0.03**	316	0.21	<2
Per capita income + LF	Social + Landscape	119.9	23.64	0.00	2	0.08	**0.04**	316	0.21	<2
NC	Landscape	130.7	34.48	-0.10	1	0.05	0.06	316	0.22	-
NC + Elevation	Landscape	130.9	34.67	-0.10	2	0.06	0.13	316	0.23	<2
LF	Landscape	130.9	34.70	-0.10	1	0.05	0.06	316	0.22	-
HDI	Social	131.1	34.91	0.16	1	0.05	0.06	316	0.23	-
Per capita income	Social	131.2	35.00	1.16	1	0.04	0.06	316	0.23	-
VT	Landscape	131.8	35.62	1.71	1	0.04	0.26	300	0.23	-
NC + LF	Landscape	132.2	35.98	0.00	2	0.05	0.15	316	0.22	<8
HDI + Per capita income	Social	132.5	36.33	0.61	2	0.05	0.15	316	0.22	<7
AC	Landscape	136.6	40.40	0.06	1	0.03	0.12	316	0.23	-
AC + UC	Landscape	136.9	40.66	0.04	2	0.04	0.25	316	0.23	<2
Elevation	Landscape	142.2	45.96	-0.10	1	0.02	0.28	316	0.23	-
UC	Landscape	146.3	50.07	0.02	1	0.00	0.63	316	0.24	-

Acronyms of predictors and other terms are as follows: ; NC: native vegetation cover; LF: area of largest forest remnant; FS: fragment size; W: open water; AC: anthropogenic habitat cover; UC: urban settlement cover; HP: human population density; NT: number of field techniques that had been deployed during mammal inventories; AS: likelihood of recording arboreal/scansorial species given the survey methods employed; VT: vegetation type; IDH: human development index; AIC: Akaike Information Criteria; Int.: model only intercept; k: number of model parameters; Sig. null: models significance; DF: degree of freedom; OD: overdispersion of models;; VIF: variance inflation factor Significant models (Sig. null) are highlighted in bold.

* Plausible “best” candidate models based on ΔAIC.

## Discussion

The majestic Atlantic Forest domain of South America once spanned over 1.1 million km^2^ and the world’s longest continuous latitudinal gradient in any tropical forest region [66]. However, over five centuries of burgeoning European settlements, the history of deforestation and degradation throughout the Atlantic Forest, in many respects, reflects the fate of all tropical forest biomes globally [16, 78]. This biome succumbed to the highest conversion rates in the highly agricultural semi-deciduous plateaus of interior regions, where forest cover is now restricted to only 7%. Regions dominated by forest conversion into cropland and cattle pastures, timber extraction, and edge effects induced by forest fragmentation currently overlap the most defaunated areas, with few representative undisturbed forest sites remaining today [16]. The last two decades showed a modest increase in forest cover across Atlantic Forest areas, consisting of secondary forests in early to mid stages of succession, compared to the 1980s [79], thereby minimally biasing our predictive proxy based on 2016 land cover data, particularly given that only 12 studies reviewed here (0.24%) were carried out before 2000. The rapidly expanding human population since the 17^th^ century, combined with the consolidation of the sugarcane, coffee, and cacao agricultural cycles of colonial and modern Brazil, left few areas intact, with wholesale losses in vertebrate species richness and biomass [80]. The contemporary Atlantic Forest domain also accommodates a disproportionately large fraction (~32.5%) of the current Brazilian population of ~208 million in less than 17% of the Brazilian territory, which clearly aggravates pressures on wild vertebrate populations.

Our analysis shows that the most defaunated regions of the Atlantic Forest are characterized by long-term post-Columbian occupation, both for agriculture and human settlements, particularly since the most intensive phase of road building and land-use conversion began in the 19^th^ century [81–83]. Currently, these regions are largely represented by severely human-modified landscapes, where remnant forest fragments continue to be eroded despite attempts to enforce environmental legislation [16]. For example, only fewer than 0.03% of all forest remnants across the Atlantic Forest are larger than 10,000 ha, with the vast majority (83.4%) of the remaining forest cover represented by patches smaller than 50 ha, which are typically embedded within an agromosaic-urban system [16] and highly accessible to hunters. To make matters worse, protected areas (PAs) across the altitudinal range covered by the Atlantic Forest is massively skewed towards elevations above 1200 masl [84], yet only less than 5% of this biome is above this threshold. Many Brazilian PAs are in fact located in the “wrong places” and less than 30% of the geographic distribution of most species coincide with PAs, thereby reducing their effectiveness [85].

Several exploitative human activities have detrimental effects that accelerate the declines of vertebrate populations, including local extinctions of large-bodied mammals [7, 86]. The main drivers of defaunation throughout the Atlantic Forest include a long and repeated history of hunting pressure, habitat conversion and fragmentation, or the synergistic combination of both (e.g. [87]). Yet, we know very little about the history of population declines induced by overhunting because baseline information on this biome is extremely scarce, not least because of the dearth of historical records from 17^th^-18^th^ century naturalists.

Considering all taxa larger than 1kg, the average species richness of Atlantic Forest mammal assemblages sampled between 1983 and 2015 was 14.7 [36]. Our results, based on the more comprehensive survey of mammal inventories carried out between 1983 and 2017 showed an average species richness of 7.1, which would represent a mean decline of 7.6 species (~51.7%) or a ~1.52% of lost species richness per year per mammal assemblage. Yet, the expected average species richness going back to historical times in Colonial Brazil would have been 25.9 species, or nearly three times higher than that of contemporary assemblages of medium- to large-bodied mammals across the Atlantic Forest. Given these historical ranges, only 23 of all 497 assemblages examined (4.62%) here retains over 70% of all species expected for any given site. These assemblages are located mainly in the Southern Atlantic province (60.8%) and were restricted to the largest forest remnants, with an average size of 10,043 ha (±9,102 ha). Another 317 (63.7%) assemblages—282 (89.9%) of which are now stranded within forest fragments smaller than 1000 ha—retain fewer than 30% of the species in their former assemblages. Almost three quarters (71.9%) of all species across all 497 Atlantic Forest sites examined here were smaller than 10 kg, compared to a historical fraction of 65.3%. Defaunation at the cluster scale was on average reduced by 22.7% compared to the assemblage scale, maintaining overall patterns for functional groups.

Even under a more conservative approach, our defaunation index was typically higher than 40%. Moreover, our arbitrary buffer in adjusting current primate distributions can also reduce the defaunation due to the large number of assemblages containing these species. These issues suggests two likely processes: (1) a methodological issue that inflates the prevalence of pseudo-absences. For example, camera-trapping studies typically reveal rare species only after from 1500 to 2000 camera trapping-days (e.g., [88]); or (2) at the metacommunity (cluster-level) scale Atlantic Forest mammals assemblages were at least partly inter-connected. Small (< 50 ha) fragments can serve as viable stepping-stones between larger fragments, increasing landscape connectivity [38] and community homogenization (e.g. decreasing beta diversity) is scale-dependent [36]. Nevertheless, our defaunation estimates, even considering clusters rather than individual sites, exceeded 50% for all functional groups, with the exception of small-bodied mammals.

Patterns of species losses across all sites are therefore disproportionately stacked against large-bodied, high trophic level species, with 73.5% of all 9,826 putative local extinctions recorded in this study represented by species larger than 10kg. Large carnivores, large frugivores and large myrmecophages were particularly heavily penalized. This directional loss in diversity introduces nonrandom impacts on the functional space of communities [7, 12–13, 32] and reinforces the notion that conservation strategies should consider all scales of any given biome. Although modern patterns of diversity may be affected by both stochastic and deterministic factors, the mammalian diversity of the Atlantic Forest, under currently prevailing conditions, depends on both the local and regional pool of species to maintain the wider patterns of diversity at the wider biogeographic scale [36].

Our results highlight concerns over the sheer scale of mammalian diversity loss across the Atlantic Forest, particularly in terms of the functional space occupied by former assemblages. The high levels of defaunation can be seen in the fact that over 57.9% of all sites recorded above-average defaunation values at the assemblage level, and reductions in density-invariant aggregate biomass to less than one quarter of former historical assemblages. These patterns of high defaunation and biomass loss were ubiquitous across all provinces, and likely resulted from the aforementioned morphoecological traits (e.g., body mass, diet), particularly among game species preferred by present or historical hunters [22–23, 89]. Several ungulates, primates and carnivores were systematically overhunted over historical timescales, and are currently extirpated at both local and provincial scales across the Atlantic Forest. Regional scale extinctions are particularly severe in the northern Atlantic Forest for several large-bodied harvest-sensitive species, including woolly spider monkeys, jaguars, white-lipped peccaries, giant armadillos, and giant anteaters, as documented by comprehensive surveys of the last forest patches throughout this ~253,000-km^2^ region [9].

Our results suggest that the systematic defaunation throughout the Atlantic Forest results in a functionally “half-empty” forest ecosystem with subsequent disruptions in the ecological roles performed by several mammal species [4, 67]. We further suggest that the Atlantic Forest of South America has faced a severe collapse in the biomass of mammals and other vertebrates, particularly primary consumers. For example, in the largest Atlantic Forest remnant (~800,000 ha), the mammal biomass declined by 98% and is 53-fold lower than in continuous neotropical forests elsewhere [6]. Jorge et al. (2013) [89] suggested that only 16% of the entire Atlantic Forest is still suitable in terms of habitat structure for the co-occurrence of the largest apex predator (*Panthera onca*), the largest herbivore (*Tapirus terrestris*), the largest seed predator (*Tayassu pecari*), and the largest arboreal seed disperser (*Brachyteles* spp.). These four species occurred in only 7.1%, 13.7%, 7.2% and 3.6% of all 497 assemblages assessed here, respectively, and they failed to jointly co-occur in any of those assemblages (which based on overlapping historical ranges should have been the case in 61 (12.3%) sites). This pattern of missing species co-occurrences could be observed across all mammalian orders. We therefore presume that the faunal integrity of small fragments, which now comprises 99.5% of all remaining fragments smaller than 1000 ha [16], is even more staggering in terms of the aggregate biomass and functional diversity of extant mammals.

Our results reinforce the detrimental effects of biome-wide deforestation and forest degradation on vertebrate species loss. Among the predictors assessed here, the overall amount of native forest cover and presence of large forest remnants (which is typically spatially associated with high elevation) attenuated the ravages of defaunation, with largely forested areas within montane regions often the most faunally intact. Both forest cover and biodiversity have decisive roles in maintaining several ecological processes [90–91] and the functional integrity of ecosystems [92]. The size distribution of forest remnants also influences both the prevalence of defaunation [9], the species richness of many taxonomic groups, and their interactions and movement patterns, prompting cascading effects that reverberate through entire ecosystems [7]. For example, tropical forests store some 55% of the global scale terrestrial carbon, and large-bodied frugivores in both Amazonian and Atlantic forests [4–5] are pivotal seed dispersal agents in maintaining between 158.6 and 736.5Mg C/km^‒2^ in aboveground carbon depending on the severity of defaunation [5].

We have shown that the ecological roles performed by medium- to large-bodied mammals within the Atlantic Forest have been heavily truncated by a pervasive extinction filter. Conservation strategies have been proposed to mitigate defaunation, including reintroduction and reinforcement efforts, assisted colonization, and rewilding [93]. Increasing the size, number and spatial spread of suitable habitat patches, particularly within protected areas, and reducing the intensity of human impacts on populations and forest habitats are obvious ways forward. Large tracts of continuous forest areas are still key preconditions for sustaining large-bodied mammal populations that cannot thrive in non-forest or hyper-fragmented landscapes, which are made worse if they are threatened by unregulated hunting [6]. Surprisingly diverse relictual assemblages of terrestrial mammals using small forest patches surrounded by sugarcane monoculture can lend some room for optimism [94], but they are often dominated by species subsidized by the agricultural matrix and many populations are unlikely to be viable in the long run. Extant mammal populations have likely persisted because they were hitherto sustained by large neighboring source populations occupying relatively large forest areas. Our study suggests that cumulative local extinctions—that can escalate into wholesale defaunation—are more likely at landscape to regional scales when nearby source populations, and their ‘rescue effect’, are missing.

Our main hypotheses outlined here have thus been largely vindicated, yet we provide a note of caution about the possible overestimation of the degree of defaunation based on differences between historical and contemporary site occupancy within the overall biome and among individual functional groups. Even under a more optimistic scenario (i.e., reducing the *a priori* measure of defaunation by 20%), the subregional biotas of the once majestic Atlantic Forest have been largely reduced to a pale shadow of their former selves. These biotas are now often severely incomplete, restricted to insufficiently large forest remnants, and trapped in an open-ended extinction vortex. The modern collapse of the Atlantic Forest mammal fauna is unprecedented in both history and pre-history and overlaps the highly deforested and degraded areas allocated to modern anthropogenic habitats. Apex-predators, megaherbivores, all large-bodied mammals, and meso- to large carnivores have all succumbed to the highest local extinction rates, but this has been buffered by large remaining forest areas along the Serra do Mar and Serra Geral montane knife-ridges. This may be easier stated than done, but we conclude that policy strategies that can maintain and/or expand forest cover remains a key conservation priority, but we realize this rests on often recalcitrant political will and robust public policies. Should these actions be implemented, it may be possible to prevent the Atlantic Forest biome from becoming an even “emptier forest” that will severely compromise patterns of diversity, ecological processes, ecosystems functioning, and ultimately human welfare.

## Supporting information

S1 File Documentation of the presumed historical occurrence of three widespread primate genera (*Alouatta*, *Brachyteles* and *Sapajus*) throughout the Atlantic Forest. (DOCX)

S2 File Database references obtained from multiple search engines describing mammal assemblage composition throughout the Atlantic Forest of South America. (DOCX)

S1 Fig Species richness of medium- to large-bodied mammals at 497 sites distributed across the Atlantic Forest of South America. Left above: Historical species richness and contemporary species richness. Inset maps show the contemporary richness of each functional group.
(PDF)
