# Supplementary material for: Wish you were here: How defaunated is the Atlantic Forest biome of its medium- to large-bodied mammal fauna?
Source: PLoS One. 2018 Sep 25;13(9):e0204515. doi: 10.1371/journal.pone.0204515 (PMC6155554; doi:10.1371/journal.pone.0204515)
Supplement: S1 File — (DOCX) [file pone.0204515.s001.docx]

**Supporting Information S1.** Documentation of presumable historical occurrence of three widespread primate genera (*Alouatta*, *Brachyteles* and *Sapajus*) throughout the Atlantic Forest.

Aguiar, L.M., Ludwig, G., Svoboda, W.K., Hilst, C.L.S., Navarro, I.T., Passos, F.C. 2007. Occurrence, local extinction and conservation of Primates in the corridor of the Upper Paraná River, with notes on other mammals. Revista Brasileira de Zoologia 24: 898-906.

Alves, L.C.P.S., Andriolo, A. 2005. Camera traps use on the mastofaunal survey of Araras Biological Reserve, IEF - RJ. Revista Brasileira de Zoociências 7: 231-246.

Antonietto, L.A., Mendes, F.D.C. 1994. São Francisco Xavier: a new site for primatological research and conservation in the Brazilian Atlantic Forest. Neotropical Primates 2: 3-4.

Araújo, R.M., Souza, M.B., Ruiz-Miranda, C.R. 2008. Densidade e tamanho populacional de mamíferos cinegéticos em duas Unidades de Conservação do Estado do Rio de Janeiro, Brasil. Iheringia Série Zoológica 98(3): 391-396.

Araújo, R.M. 2009. Ocorrência e densidade populacional de bugio (*Alouatta guariba* Lacépède, 1799) e macaco-prego (*Cebus nigritus* Erxleben, 1777) em fragmentos de Mata Atlântica no estado do Rio de Janeiro. MSc. Thesis, Universidade Estadual do Norte Fluminense.

Cáceres, N.C., Nápolib, R.P., Casellac, J., Hannibald, W. 2010. Mammals in a fragmented savannah landscape in south-western Brazil. Journal of Natural History 44: 481-502.

Canale, G.R., Guidorizzi, C.E., Kierulff, M.C., Gatto, C.A. 2009. First record of tool use by wild populations of the yellow-breasted capuchin monkey (*Cebus xanthosternos*) and new records for the bearded capuchin (*Cebus libidinosus*).

Canale, G.R. unpublished data.

Chiarello, A.G. 1999. Effects of fragmentation of the Atlantic forest on mammal communities in southeastern Brazil. Biological Conservation 89: 71-82.

Chiarello, A.G. 2000. Conservation value of a native forest fragment in a region of extensive agriculture. Revista Brasileira de Biologia 60 (2): 237-247.

Chiarello, A.G. Melo, F.R. 2001. Primate population densities and sizes in Atlantic forest remnants of northern Espírito Santo, Brazil. International Journal of Primatology 22 (3): 379-396.

Chiarello, A.G. 2003. Primates of the Brazilian Atlantic forest: the influence of forest fragmentation on survival. Primates in Fragments: Ecology and Conservation (ed. by L.K. Marsh), pp. 99-121. Kluwer Academic/Plenum Publishers, New York, USA.

Cullen-Jr, L., Bodmer, E.R., Valladares-Padua, C. 2001. Ecological consequences of hunting in Atlantic forest patches, São Paulo, Brazil. Oryx 35: 137-144.

Cunha, A.A., Vieira, M.V. 2004. Present and past primate community of the Tijuca forest, Rio de Janeiro, Brasil. Neotropical Primates 12: 153-154.

Dias, L.G., Strier, K.B. 2000. Agonistic encounters between muriquis *Brachyteles arachnoides hypoxanthus* (Primates, Cebidae), and other animals at the Estação Biológica de Caratinga, Minas Gerais, Brazil. Neotropical Primates 8: 138-140.

Fialho, M.S., Gonçalves, G.F. 2008. Primatas da RPPN Gargaú, Paraíba, Brasil. Neotropical Primates 15(2): 50-54.

Flesher, K.M. 2006. The Biogeography of the Medium and Large Mammals in a Human-dominated Landscape in the Atlantic Forest of Bahia, Brazil: Evidence for the Role of Agroforestry Systems as Wildlife Habitat. PhD Thesis, Rutgers University.

Fortes, V.B. 2008. Ecologia e comportamento do bugio-ruivo (*Alouatta guariba clamitans* Cabrera, 1940) em fragmentos florestais na depressão central do Rio Grande do Sul, Brasil. PhD Thesis, Pontifícia Universidade Católica do Rio Grande do Sul.

Galetti, M. et al., 2009. Priority areas for the conservation of Atlantic forest large mammals. Biological Conservation 142: 1229-1241.

Gheler-Costa, C., Verdade, L.M., Almeida, A.F. 2002. Mamíferos não-voadores do campus "Luiz de Queiroz", Universidade de São Paulo, Piracicaba, Brasil. Revista Brasileira de Zoologia 19: 203-214.

González-Solís, J., Guix, J., Mateos, E., Llorens, L. 2001. Population density of primates in a large fragment of the Brazilian Atlantic rainforest. Biodiversity & Conservation 10: 1267-1282.

Jardim, M.M.A. 2005. Ecologia populacional de bugios-ruivos (*Alouatta guariba*) nos municípios de Porto Alegre e Viamão, RS, Brasil. PhD Thesis, Universidade Estadual de Campinas.

Loretto, D., Rajão, H. 2005. Novos registros de primatas no Parque Nacional do Itatiaia, com ênfase em *Brachyteles arachnoides* (Primates, Atelidae). Neotropical Primates 13(2): 28-30.

Martins, M.M. 2005. Density of primates in four semi-deciduous forest fragments of São Paulo, Brazil. Biodiversity & Conservation 14: 2321-2329.

Melo, F. R. 2004. Primatas e áreas prioritárias para a conservação da biodiversidade no vale do rio Jequitinhonha, Minas Gerais. PhD, Universidade Federal de Minas Gerais.

Moreira, L.L.B. 2009. Primatas das serras da Lontras e Javi: estado das populações e seu papel na conservação regional da comunidade de primatas no sul da Bahia. MSc. Thesis, Universidade Estadual de Santa Cruz.

Neves, L.G. 2008. Distribuição geográfica e conservação de *Callithrix kuhlii* (Coimbra-Filho, 1985) (Primates, Callitrichidae) no sul da Bahia, Brasil. MSc. Thesis, Universidade Estadual de Santa Cruz.

Oliveira, M.F., Manzatti, L. 1996. New location for the muriqui (*Brachyteles arachnoides*) in the State of São Paulo, Brazil. Neotropical Primates 4(3): 84-84.

Passamani, M. 2008. Densidade e tamanho de grupo de primatas na Mata Atlântica serrana do sudoeste do Espírito Santo. Revista Brasileira de Zoociências 10(1): 29-34.

Ribeiro, S., Bicca-Marques, J.C. 2005. Landscape characteristics and their influence on the occurrence of brown howling monkeys (*Alouatta guariba clamitans* Cabrera, 1940; Primates, Atelidae) in forest fragments in the Vale do Taquari, RS - Brazil. Natureza & Conservação 3: 168-181.

Trevellin, L.C., Port-Carvalho, M., Silveira, M., Morell, E. 2007. Abundance, habitat use and diet of *Callicebus nigrifrons* Spix (Primates, Pitheciidae) in Cantareira State Park, São Paulo, Brazil. Revista Brasileira de Zoologia 24: 1071-1077.

Villar, D. 2006. Censo e Ecologia Comportamental de Macaco-prego – *Cebus libidinosus* - em área de cerrado do Parque Estadual Altamiro de Moura Pacheco. Msc Thesis, Universidade Católica de Goiás.
