# Supplementary material for: Wish you were here: How defaunated is the Atlantic Forest biome of its medium- to large-bodied mammal fauna?
Source: PLoS One. 2018 Sep 25;13(9):e0204515. doi: 10.1371/journal.pone.0204515 (PMC6155554; doi:10.1371/journal.pone.0204515)
Supplement: S2 File — (DOCX) [file pone.0204515.s002.docx]

**Supporting Information S3.** Database references obtained from multiple search engines describing mammal assemblage composition throughout the Atlantic Forest of South America.

Abreu-Júnior, E.F. and Köhler, A. (2009). Mammalian fauna of medium and large sized in the RPPN of UNISC, RS, Brazil. Biota Neotrop. 9, 169–174.

Alves, T. R., Fonseca, R. C., and Engel, V. L. (2012). Mamíferos de médio e grande porte e sua relação com o mosaico de habitats na cuesta de Botucatu, Estado de São Paulo, Brasil. Iheringia. S. Zool. 102, 150-158.

Albuquerque, H.G., Martins, P.F., Pessôa, F.S., Carvalho, T., Modesto, T.C., Luz, J.J., Raíces, D.S.L., Ardete, N.C., Lessa, I.C.M., Attias, N., Jordão-Nogueira, T., Enrici, M.C. and Bergallo, H.G. (2013). Mammals of a forest fragment in Cambuci municipality, state of Rio de Janeiro, Brazil. Check List. 9, 1505-1509.Alves, T.R., Fonseca, R.C.B. and Engel, V.L. (2012). Medium and large sized mammalians and their relation to habitat patches at the Botucatu cuesta, state of São Paulo, Brazil. Iheringia. Iheringia Sér. Zool. 102, 150-158.

Bogoni, J.A., Bogoni, T.C., Graipel, M.E. and Marinho, J.R. (2013). The Influence of Landscape and Microhabitat on the Diversity of Large-and Medium-Sized Mammals in Atlantic Forest Remnants in a Matrix of Agroecosystem and Silviculture. ISRN Forestry 2013.

Bogoni, J.A., Cherem, J.J., Giehl, E.L.H., Oliveira-Santos, L.G.R., Castilho, P.V., Picinatto-Filho, V., Fanticini, F.M., Tortato, M.A., Luiz, M.R., Rizzaro, R. and Graipel, M.E. (2016a). Landscape features lead to shifts in communities of medium to large-bodied mammals in subtropical Atlantic Forest. J. Mammal., gyv215.in press.

Bogoni, J.A., Graipel, M.E., Castilho, P.V., Fantacini, F.M., Kuhnen, V.V., Luiz, M.R., Maccarini, T.B., Marcon, C.B., Teixeira, C.S.P., Tortato, M.A., Vaz-de-Mello, F.Z., Hernández, M.I.M (2016b). Contributions of the mammal community, habitat structure, and spatial distance to dung beetle community structure. Biod. Cons. 25, 1661-1675.

Bovendorp, R. S. and Galetti, M. (2007). Density and population size of mammals introduced on a land-bridge island in southeastern Brazil. Biol. Invasions 9, 353-357.

Brocardo, C.R. and Cândido-Júnior, J.F. (2012). Persistência de mamíferos de médio e grande porte em fragmentos de Floresta Ombrófila Mista no estado do Paraná, Brasil. Rev. Árvore 36, 301-310.

Carvalho I.D., Oliveira R. and Pires A.S. (2014) Medium and large-sized mammals of the Reserva Ecológica de Guapiaçú, Cachoeiras de Macacu, RJ. Biota Neotrop. 14, 1–9.

Carvalho, W.D., Godoy, M.S.M., Adania, C.H. and Esbérard, C.E.L. (2013). Non-volant mammal assemblage of serra do Japi biological reserve, Jundiaí, São Paulo, southeastern Brazil. Bioscience J. 29, 1370-187.

Cassano, C.R., Barlow, J. and Pardini, R. (2012). Large mammals in an agroforestry mosaic in the Brazilian Atlantic Forest. Biotropica 44, 818-825.

Chagas, R. R. D., Junior, E. M. S., Souza-Alves, J. P. and Ferrari, S. F. (2011). Fazenda Trapsa, um refúgio de diversidade de mamíferos de médio e grande porte em Sergipe, Nordeste do Brasil. Rev. Nordestina. Biol. 19, 35-43.Chagas.et.al.2012

Cherem, J.J. and Perez, D.M. (1996). Mamíferos terrestres de floresta de araucária no município de Três Barras, Santa Catarina, Brasil. Biotemas 9, 29-46.

Cherem, J.J., Graipel, M.E., Tortato, M.A., Althoff, S.L., Brüggemann, F., Matos, J.Z., Voltolini, J.C., Freitas, R.R., Illenseer, R., Hoffmann, F., Ghizoni-Jr., I.R., Bevilacqua, A., Reinicke, R., Oliveira, C.H.S., Filippini, A., Furnari, N., Abati, K., Moraes, M., Moreira, T.T., Oliveira-Santos, L.G.R., Kuhnen, V.V., Maccarini, T.B., Goulart, F.V.B., Mozerle, H.B., Fantacini, F.M., Dias, D., Penedo-Ferreira, R., Vieira, B.P. and Simões-Lopes, P.C. (2011). Mastofauna terrestre do Parque Estadual da Serra do Tabuleiro, Estado de Santa Catarina, sul do Brasil. Biotemas 24, 73-84.

Chiarello, A.G. (1999). Effects of fragmentation of the Atlantic forest on mammal communities in South-eastern Brazil. Biol. Conserv. 89, 71–82.

Cullen Jr., L., Bodmer, R.E. and Valladares-Pádua, C. (2001). Ecological consequences of hunting in Atlantic forest patches, São Paulo, Brazil. Oryx 35, 137–144.

Cunha, A. A. (2010). Negative effects of tourism in a Brazilian Atlantic Forest National Park. J. Nat. Conserv. 18, 291-295.

Cunha, A.A., and Rajão, H. (2007). Mamíferos Terrestres e Aves da Terra Indígena Sapukai (Aldeia Guarani do Bracui), Angra dos reis, Rj, Brasil. Bol. Mus. Biol. Mello Leitão 21, 19-34.

Delciellos, A.C., Novaes, R.L.M., Loguercio, M.F.C., Geise, L., Santori, R.T., Souza, R.F.S., Papi, B.S., Raíces, D., Vieira, N.R., Feliz, S., Detogne, N., Silva, C.C.S., Bergallo, H.G. and Rocha-Barbosa, O. (2012). Mammals of Serra da Bocaina National Park, state of Rio de Janeiro, southeastern Brazil. Check List 8, 675-692.

Dias, W.A.F., Tezori, R.F.F. and Oliveira, A.K. (2012). Registro de mamíferos de médio e grande porte em dois fragmentos florestais no município de São Carlos, Estado de São Paulo. Multiciêcia 11, 277-293.

Di Bitetti, M.S., Paviolo, A. and De Angelo, C. (2014). Camera trap photographic rates on roads vs. off roads: location does matter. Mastozool. Neotrop. 21, 37-46.

Dotta, G. and Verdade, L.M. (2011). Medium to large-sized mammals in agricultural landscapes of south-eastern Brazil. Mammalia 75, 345-352.

Duprat, P.L. and Andriolo, A. (20132011). Mastofauna não-voadora de médio e grande porte em um fragmento de Mata Atlântica no município de Rio Novo, MG. Rev. Bras. Zoociênc. 13, 163-172.

Eduardo, A.A. (2011). Spatial patterns of mammalian diversity in a fragmented landscape in southeastern Brazil. Rev. Bras. Biociênc. 9, 252-255.

Espartosa, K.D., Pinotti, B.T. and Pardini, R. (2011). Performance of camera trapping and track counts for surveying large mammals in rainforest remnants. Biodiver. Conserv. 20, 2815-2829.

Falcão, F.C., Guanaes, D.H.A. and Paglia, A. (2012). Medium and large-sized mammals of RPPN Estação Veracel, southernmost Bahia, Brazil. Check List 8, 929-934.

Flesher, K.M. and Laufer, J. (2013). Protecting wildlife in a heavily hunted biodiversity hotspot: a case study from the Atlantic Forest of Bahia, Brazil. Trop. Conserv. Sci. 6, 181-200.

Fornitano, L., Angeli, T., Costa, R.T., Olifiers, N. and Bianchi, R.C. (2015). Medium to large-sized mammals of the Augusto Ruschi Biological Reserve, São Paulo State, Brazil. Oecologia Oecol . Australis 19, 232-243.

Gatti, A., Segatto, B., Carnelli, C.C. and Moreira, D.O. (2014). Mamíferos de médio e grande porte da Reserva Biológica Augusto Ruschi, Espírito Santo. Natureza on line 12, 61-68.

Gatti, A., Ferreira, P.M., Cunha, C.J., Seibert, J.B., Moreira, D.O. 2017. Diversity of medium-sized and large mammals from Atlantic Forest remnants in southern Minas Gerais state, Brazil. Oecologia Australis 21(2): 171-181.

Geise, L., Pereira, L.G., Bossi, D.E.P. and Bergallo, H.G. (2004). Pattern of elevational distribution and richness of non volant mammals in Itatiaia National Park and its surroundings, in southeastern Brazil. Braz. J. Biol. 64, 599-612.

Giacomini, H.C. and Galetti, M. (2013). An index for defaunation. Biol. Conserv. 163, 33-41.

Gomes-Albuquerque, H., Martins, P.F., Pessôa, F.S., Modesto, T.C., Luz, J.L., Raíces, D.S., Ardente, N.C., Lessa, I.C.M, Attias, N., Jordão-Nogueira, T., Enrici, M. C. and Bergallo, H.G. (2013). Mammals of a forest fragment in Cambuci municipality, state of Rio de Janeiro, Brazil. Check List 9, 1505-1509.

Graipel, M.E., Cherem, J.J. and Ximienez, A. (2001). Mamíferos terrestres não voadores da Ilha de Santa Catarina, sul do Brasil. Biotemas 14, 109-140.

Hendges, C.D., Salvador, C.H. and Nichele, M.A. (2015). Mamíferos de médio e grande porte de remanescentes de Floresta Estacional Decidual no Parque Estadual Fritz Plaumann e em áreas adjacentes, Sul do Brasil. Biotemas 28, 121-134.

Junges, S.O. and Cademartori, C.V. (2012). Composição da mastofauna de médio e grande porte em um remanescente de floresta com araucária no sul do Brasil. Mouseion 13, 170-181.

Juraszek, A., Bazilio, S. and Golec, C. (2014). Levantamento de mamíferos de médio e grande porte na RPPN Federal Corredor do Iguaçu na região centro-oeste do Paraná. Acta Iguazu 3, 113-123.

Kasper, C.B., Feldens, M.J., Mazim, F.D., Schneider, A., Cademartori, C.V. and Grillo, H.C.Z. (2007a). Mamíferos do Vale do Taquari, região central do Rio Grande do Sul. Biociências (On-line) 15, 53-62.

Kasper, C.B., Mazim, F.D., Soares, J.B.G., Oliveira, T.G. and Fabián M.E. (2007b). Composição e abundância relativa dos mamíferos de médio e grande porte no Parque Estadual do Turvo, Rio Grande do Sul, Brasil. Rev. Brasil. Zool. 24, 1087-1100.

Lima, J.C.S. and Pasciani, V. (2014). Riqueza de espécies de mamíferos de médio e grande porte na Fundação Jardim Botânico de Poços de Caldas, Minas Gerais, Brasil. Rev. Biociências. 20, 62-70.

Machado, F.S., Almeida, A.F., Barros, D.A., Pereira, J.A.A., Silva, R.S., Pereira, A.A.S. 2016. Diversity of medium-sized and large mammals from Atlantic Forest remnants in southern Minas Gerais state, Brazil. Check List 12(5): 1962. DOI: 10.15560/12.5.1962.

Maciel, L. and Maciel, K.P.W.A. (2015). Levantamento preliminar de mamíferos silvestres em uma área de Floresta Ombrófila Mista na região de Porto Vitória-PR. Rev. Elet. Biol. 8, 13-28.

Magioli, M., Ferraz, K.M.P.M.B. and Rodrigues, M.G. (2014). Medium and large-sized mammals of an isolated Atlantic Forest remnant, southeast São Paulo State, Brazil. Check List 10, 850-856.

Marques, R.V., Cademartori, C.V. and Pacheco, S.M. (2011). Mastofauna no Planalto das Araucárias, Rio Grande do Sul, Brasil. Rev. Bras. Biociênc. 9, 278-288.

Martins, T.O., Bunhuolo, S.P., Ortêncio, H., Lacher, T.E. 2016. Large and medium-sized mammals in the urban park Cinturão Verde, Cianorte, northwestern Paraná. Check List 12(2): 1851. DOI: 10.15560/12.2.1851.

Mendes, C.L.S., Santos, B.O., Laia, W.P. and Souza, L.A. (2014). Diversidade de mamíferos de médio e grande porte da reserva particular do patrimônio natural da Mata do Sossego e seu entorno, Minas Gerais. Rev. Bras. Zoociênc. 16, 27-41.

Mendes-Pontes, A.R., Beltrão A.C.M, Normande I.C., Malta A.J.R., Silva Júnior, A.P., Santos, A.M.M. 2016. Mass Extinction and the Disappearance of Unknown Mammal Species: Scenario and Perspectives of a Biodiversity Hotspot’s Hotspot. PLoS ONE 11(5): e0150887. DOI:10.1371/journal.pone.0150887.

Miranda, J.M.D., Rios, R.F.M. and Passos, F.C. (2008). Contribuição ao conhecimento dos mamíferos dos Campos de Palmas, Paraná, Brasil. Biotemas 21, 97-103.

Modesto, T.C., Pessôa, F.S., Enrici, M.C., Attias, N., Jordão-Nogueira, T., Costa, L.M., Albuquerque, H.C. and Bergallo, H.G. (2008). Mamíferos do Parque Estadual do Desengano, Rio de Janeiro, Brasil. Biota Neotrop. 8, 153-159.

Morcatty, T.Q., El Bizri, H.R., Carneiro, H.C.S., Biasizzo, R.L., Alméri, C.R.O., Silva, E. S., Rodrigues, F.H.G., and Figueira, J.E.C. (2013). Habitat loss and mammalian extinction patterns: are the reserves in the Quadrilátero Ferrífero, southeastern Brazil, effective in conserving mammals?. Ecol. Res. 28, 935-947.

Negrão, M.D.F.F. and Valladares-Pádua, C. (2006). Registros de mamíferos de maior porte na Reserva Florestal do Morro Grande, São Paulo. Biota Neotrop. 6, 1-13.

Norris, D., Ramírez, J.M., Zacchi, C. and Galetti, M. (2012). A Survey of mid and large bodied mammals in Núcleo Caraguatatuba, Serra do Mar State Park, Brazil. Biota Neotrop. 12, 127-133.

Nunes, A.V., Scoss, L.M., Prado, M.R. and Lessa, G.M. (2013). Survey of large and medium-sized terrestrial mammals in the Serra do Brigadeiro State Park, Minas Gerais, Brazil. Check List 9, 240-245.

Oliveira, L.P., Aguiar, D., Margarido, T.C.C. and Pachaly, J.R. (2013). Caracterização faunística de mamíferos de médio e grande porte de um fragmento florestal do noroeste do estado do Paraná, Brasil. Arq. Ciênc. Vet. Zool. UNIPAR 15, 109-114.

Oliveira, V.B., Linares, A.M., Castro-Corrêa, G.L. and Chiarello, A.G. (2013). Inventory of medium and large-sized mammals from Serra do Brigadeiro and Rio Preto State Parks, Minas Gerais, southeastern Brazil. Check List 9, 912-919.

Paglia, A.P., Perini, F.A., Lopes, M.O. and Palmuti, C.F. (2005). Novo registro de Blarinomys breviceps (Winge, 1888) (Rodentia, Sigmodontinae) no estado de Minas Gerais, Brasil. Lundiana 6, 155-157.

Passamani, M., Jenilson, D. and Lopes, S.A. (2005). Mamíferos não-voadores em áreas com predomínio de Mata Atlântica da Samarco Mineração SA, município de Anchieta, Espírito Santo. Biotemas 18, 135-149.

Passamani, M., Mendes, S.L. and Chiarello, A.G. (2000). Non-volant mammals of the Estação Biológica de Santa Lúcia and adjacent areas of Santa Teresa, Espírito Santo, Brazil. Bol. Mus. Biol. Mello Leitão 11, 201-214.

Penido, G. and Zanzini, A.C.S. (2012). Checklist of large and medium-sized mammals of the Estação Ecológica Mata do Cedro, an Atlantic forest remnant of central Minas Gerais, Brazil. Check List 8, 712-717.

Penter, C., Fabián, M.E. and Hartz, S.M. (2008). Inventário rápido da fauna de mamíferos do Morro Santana, Porto Alegre, RS. Rev. Bras. Biociênc. 6, 117-125.

Percequillo, A., Santos, K., Campos, B., Santos, R., Toledo, G. and Langguth, A. (2007). Mamíferos dos remanescentes florestais de João Pessoa, Paraíba. Biol. Geral Exp. 7, 17-31.

Pereira, S.N., Dias, D., Lima, I.P., Maas, A.C.S., Martins, M. A., Bolzan, D.P., França, D.S., Oliveira, M.B., Peracchi, A.L. and Ferreira, M.F. (2013). Mamíferos de um Fragmento Florestal em Volta Redonda, Estado do Rio de Janeiro= Mammals of a forest fragment in Volta Redonda, Rio de Janeiro state. Bioscience J. 29, 1017-1027.

Peters, F.B., Roth, P.R.O., Machado, L.F., Coelho, E.L., Jung, D.M.H. and Christoff, A.U. (2010). Assembléia de mamíferos dos agroecossistemas constituintes da bacia hidrográfi ca do rio da Várzea, Rio Grande do Sul. Biotemas 23, 91-107.

Pires, D.P.S. and Cademartori, C.V. (2012). Medium and large sized mammals of a semideciduous forest remnant in southern Brazil. Biota Neotrop. 12, 239-245.

Portella, T.P. and Flynn, M.N. (2012). Inventário rápido de mamíferos de médio e grande porte da Área de Proteção Ambiental da Ilha Comprida, SP. RevInter Revista Intertox de Toxicologia, Risco Ambiental e Sociedade 5, 19-37.

Prado, H.M., Murrieta, R.S.S., Adams, C. and Brondizio, E.S. (2014). Local and scientific knowledge for assessing the use of fallows and mature forest by large mammals in SE Brazil: identifying singularities in folkecology. J. Ethnobiol. Ethnomed. 10.

Prado, M.R., Rocha, E.C. and Del Giudice, G.M.L. (2008). Medium and large-sized mammal in a forest fragment of atlantic forest, Minas Gerais, Brazil. Rev. Árvore Arv. 32, 741-749.

Preuss, J.F., Pfeifer, G.B., Toral, J.F., Bressan, S.J. 2016. Levantamento Rápido de Mamíferos Terrestres em Um Remanescente de Mata Atlântica do Sul do Brasil. Unoesc & Ciência 7(1): 89-86.

Reale, R., Fonseca, R.C.B. and Uieda, W. (2014). Medium and Large-sized Mammals in a Private Reserve of Natural Heritage in the Municipality of Jaú, São Paulo, Brazil. Check List 10, 997-1004.

Rocha, E.C., Soares, K.L. and Pereira, I.M. (2015). Medium-and large-sized mammals in Mata Atlântica State Park, southeastern Goiás, Brazil. Check List 11, 1802.

Rocha-Mendes, F., Mikich, S.B., Bianconi, G.V. and Pedro, W.A. (2005). Mammals of the municipality of Fenix, Parana, Brazil: ethnozoology and conservation. Rev. Bras. Zool. 22, 991-1002.

Rosa, C.A., Souza, A.C. 2017. Large and medium-sized mammals of Nova Baden State Park, Minas Gerais, Brazil Check List 13(3): 2141. DOI: 10.15560/13.3.2141.

Rossaneis, B.K. (2014). Mamíferos de médio e grande porte em pequenos remanecentes florestais da mata atlântica com influências antropogênicas no norte do Paraná. Semina, 35, 15-24.

Santos, K.K., Pacheco, G.S.M., Passamani, M. 2016. Medium-sized and large mammals from Quedas do Rio Bonito Ecological Park, Minas Gerais, Brazil. Check List 12(1): 1830. DOI: 10.15560/12.1.1830.

Sousa, M.A.N. and Gonçalves, M.F. (2004). Mastofauna terrestre de algumas áreas sobre influência da Linha de Transmissão (LT) 230 KV PE/PB, CIRCUITO 3. Revista Rev. de Biologia Bio.e Ciências Ciên.da Terra Ter. 4, 1-14.

Spezia, M.B., Grasel, D. and Miranda, G. (2013). Inventário rápido de mamíferos não voadores em um fragmento florestal do bioma Mata Atlântica. Unoesc and Ciência-. ACBS 4, 145-154

Srbek-Araujo, A.C., Kierulff, M.C.M. Mamíferos de Médio e Grande Porte das Florestas de Tabuleiro do Norte do Espírito Santo: Grupos Funcionais e Principais Ameaças. In: Rolim, S.G.; Menezes, L.F.T., Srbek-Araujo, A.C. (eds.). Floresta Atlântica de Tabuleiro: diversidade e endemismos na Reserva Natural Vale. 496p

Stallings, J.R., Fonseca, G.A.B., Pinto, L.P.S., Aguiar, L.M.S. and Sábato, E.L. (1991). Mamíferos do Parque Florestal Estadual do Rio Doce, Minas Gerais, Brasil. Rev. Bras. Zool. 7, 663-77.

Tortato, F.R., Testoni, A.F. and Althoff, S.L. (2014). Mastofauna terrestre da Reserva Biológica Estadual do Sassafrás, Doutor Pedrinho, Santa Catarina, Sul do Brasil. Biotemas 27, 123-129.

Wallauer, J.P., Becker, M., Martins-Sá, L.G., Liermann, L.M., Perretto, S.H. and Schermack, V. (2000). Levantamento dos mamíferos da Floresta Nacional de Três Barras-Santa Catarina. Biotemas 13, 103-127.

Wolfart, M.R., Da Fré, M., Miranda, G.B. and Lucas, E.M. (2013). Mamíferos terrestres em um remanescente de Mata Atlântica, Paraná, Brasil. Biotemas 26, 111-119.
