# Supplementary figures and images for: Wish you were here: How defaunated is the Atlantic Forest biome of its medium- to large-bodied mammal fauna?
Source: PLoS One. 2018 Sep 25;13(9):e0204515. doi: 10.1371/journal.pone.0204515 (PMC6155554; doi:10.1371/journal.pone.0204515)

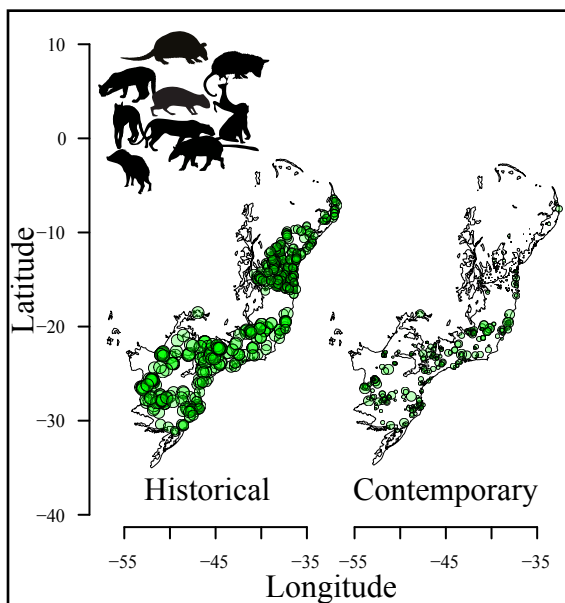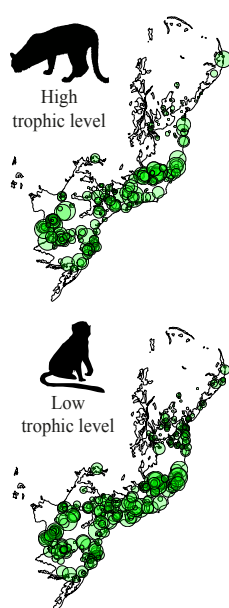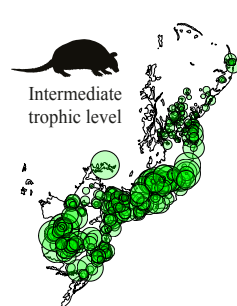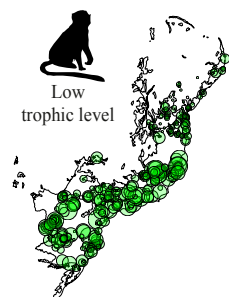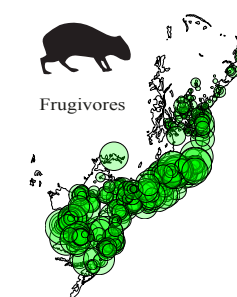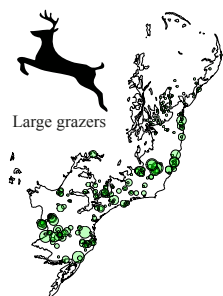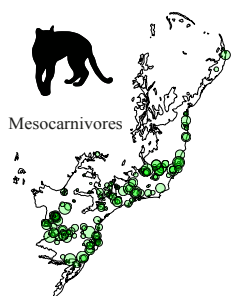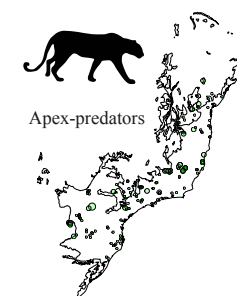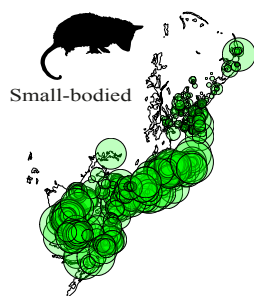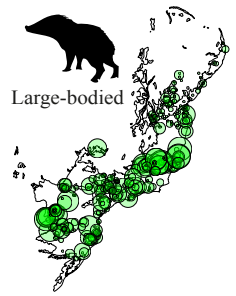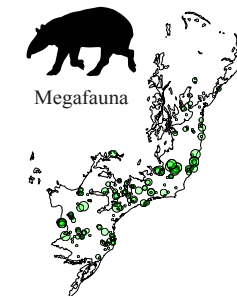

Supplement: S1 Fig — Left above: Historical species richness and contemporary species richness. Inset maps show the contemporary richness of each functional group. (PDF) [file pone.0204515.s003.pdf]
